# Characterization of Anti-Cancer Activities of Violacein: Actions on Tumor Cells and the Tumor Microenvironment

**DOI:** 10.3389/fonc.2022.872223

**Published:** 2022-05-11

**Authors:** Charlotte Dahlem, Shilpee Chanda, Jan Hemmer, Hanna S. Schymik, Michael Kohlstedt, Christoph Wittmann, Alexandra K. Kiemer

**Affiliations:** ^1^ Pharmaceutical Biology, Department of Pharmacy, Saarland University, Saarbruecken, Germany; ^2^ Institute of Systems Biotechnology, Saarland University, Saarbruecken, Germany

**Keywords:** natural compounds, immunogenicity, chemoresistance, reactive oxygen species, IL6, TNF, IL1β, iNOS

## Abstract

Natural products have been shown to serve as promising starting points for novel anti-cancer drugs. In this study, the anti-cancer activities of the purple compound violacein, initially isolated from *Chromobacterium violaceum*, were investigated. To highlight the crucial role of the tumor microenvironment on the effectiveness of cancer therapies, this study includes effects on macrophages as prototypic cells of the microenvironment in addition to the investigation of tumor-centric activities. Using 2D and 3D cell culture models, automated live-cell microscopy, and biochemical analyses, violacein was demonstrated to inhibit tumor cell proliferation and migration. The violacein-triggered tumor cell death was further associated with caspase 3-like activation and ATP release. Stimuli released from dead cells resulted in inflammatory activation of macrophages, as shown by NF-κB reporter cell assays, macrophage morphology, and gene expression analysis. Moreover, macrophages deficient in the inflammasome component *Nlrp3* were found to be significantly less sensitive towards treatment with violacein and doxorubicin. Taken together, this study provides new insights into the biological activity of violacein against cancer. In addition, the *in vitro* data suggest immunogenic features of induced cell death, making violacein an interesting candidate for further studies investigating the compound as an inducer of immunogenic cell death.

## 1 Introduction

In the past, natural products have played a key role in the discovery of new cancer drugs (1). The natural compound violacein was initially isolated from *Chromobacterium violaceum* before metabolic engineering offered the possibility for more productive isolation, e.g., using *Escherichia coli* as a platform (2, 3). The anti-cancer activity of the purple compound was first described in the early 2000s. Besides that, various actions have been supposed, among them antibacterial, antiviral, and antifungal activities (3, 4). Being equipped with this broad spectrum of potential applications, violacein has attracted interest in understanding its mechanism of action.

In the context of its anti-cancer activity, diverse actions of violacein against different hallmarks of cancer have been reported. This includes but is not limited to the induction of cell death, inhibition of proliferation and migration, and the suppression of cancer stemness (5–8). The application of violacein in *in vivo* mouse models of acute and chronic inflammation demonstrated anti-inflammatory effects, involving a decreased production of inflammatory cytokines (9). In contrast, the *in vitro* treatment of murine and human macrophages resulted in their inflammatory activation *via* TLR8 signaling (10).

Macrophages display a broad polarization spectrum fulfilling diverse functions depending on the needs of their specific environment (11). The polarization stages referred to as M1 and M2 polarizations mark the extreme ends of this spectrum, with the transition between them being seamless. However, inflammatory M1 macrophages predominantly play a role in host defense, while M2 macrophages are associated with the resolution of inflammation and tissue repair. Macrophage phenotypes are characterized by a variety of surface and intracellular receptors, multiple signal transduction pathways, and adaptable arrays of gene expression (12, 13). As one example, expression of the inducible nitric oxide synthase (iNOS) has been described as a hallmark of M1 macrophages, producing NO as an inactivating and destroying infectious agent (14).

In the tumor microenvironment (TME), tumor-associated macrophages (TAMs) show a high presence and are key players linking cancer progression and inflammation. While inflammatory M1 macrophages suppress tumor growth, anti-inflammatory M2 macrophages rather promote disease progression by facilitating tumor proliferation, migration, and angiogenesis (15). In line with their tumor-promoting functions, a correlation between an increased presence of M2-like TAMs and poor prognosis has been found in various tumor entities (16), making them an attractive target in the TME.

Since cells of the TME, such as macrophages, have been shown to influence the effectiveness of cancer therapeutics significantly, successful therapeutic strategies against cancer involve more than just killing malignant cells (17, 18). Besides direct actions of the drug targeting immune cells in the TME, cancer cell death can be immunogenic or nonimmunogenic.

For instance, cells that undergo apoptosis are engulfed by phagocytes in an immunologically “silent” manner, leading to active suppression of the immune response (19). Moreover, the phagocytosis of the cellular corpses fosters macrophages into an M2 phenotype, thereby further promoting tumor growth (20). In contrast, there is the concept of immunogenic cell death (ICD), which triggers an immune response against antigens of dead cells (21). While *in vivo* vaccination experiments still represent the gold standard in detecting potential ICD inducers, distinct properties of ICD have been revealed. One of them is the pre-apoptotic exposure of calreticulin (CRT) at the surface of the dying cells, labeling them for phagocytosis, as well as the release of ATP and the nuclear high mobility group box 1 (HMGB1) protein. While HMGB1 activates toll-like receptor 4 (TLR4) and subsequently myeloid cells, ATP binds purinergic P2RX7 receptors, stimulating the nucleotide-binding oligomerization domain-like receptor family, pyrin domain–containing 3 (NLRP3) inflammasome to produce interleukin-1β (IL1β), stimulating T lymphocytes against tumor-specific antigens (22, 23).

## 2 Materials and Methods

### 2.1 Materials

Coenzyme A (Sigma #C3019), D-luciferin (Cayman #Cay25836), luciferase (Sigma #SRE0045), HRP (Peroxidase from horseradish, Type II, Sigma #P8250-5KU), HVA (Homovanillic acid, Sigma #H1252). Other chemicals were obtained from Sigma-Aldrich unless stated otherwise.

### 2.2 Cell Culture

Huh7 and A549 were cultured in RPMI-1640, while HCT116, PANC-1, SW620, MCF7, SK-MEL-5, HeLa, CC-SW-1, and HepG2 cells were cultured in DMEM. Media was supplemented with 10% FCS, 100 U/ml penicillin/streptomycin, and 2 mM glutamine. Media and supplements were purchased from Sigma-Aldrich (#R0883, #D6546, #F7524, #P433, #G7513). All cell lines were maintained at 37°C and in 5% CO_2_.

#### 2.2.1 Reporter Cells

RAW-Blue™ cells (In vivoGen) were grown in DMEM medium supplemented with 10% heat-inactivated FCS (30 min at 56°C), 100 U/ml penicillin/streptomycin, 2 mM glutamine, 100 μg/ml Normocin, and 200 μg/ml Zeocin for selection.

THP1-XBlue™ cells (In vivoGen) were grown in RPMI-1640 supplemented with 10% heat-inactivated FCS (30 min at 56°C), 100 U/ml penicillin/streptomycin, 2 mM glutamine, 100 μg/ml Normocin, and 200 μg/ml Zeocin for selection. For macrophage differentiation, 50,000 cells per well were cultured in 96 well plates in the presence of 30 ng/ml phorbol 12-myristate 13-acetate (PMA, Calbiochem #524400) for 48 hours.

HEK-Blue™ hTLR4 cells and HEK-Blue™ Null2 cells (In vivoGen) were grown in DMEM supplemented with 10% heat-inactivated FCS (30 min at 56°C), 100 U/ml penicillin/streptomycin, 2 mM glutamine, 100 μg/ml Normocin, and 1x HEK-Blue™ Selection.

#### 2.2.2 3D Cell Culture

96 well plates were coated with 50 μl sterile 1.5% agarose. 3,000 HCT116 cells were seeded on agarose-coated plates and centrifuged for 5 min at 250g. Spheroids were grown for 6 days before they were treated with violacein or DMSO solvent control.

#### 2.2.3 Doxorubicin-Resistant Huh7

Doxorubicin-resistant Huh7 cells were generated as described previously (24) and cultured in RPMI-1640 containing 2 μM doxorubicin (Alfa Aesar #J- 64000). Cells were seeded in RPMI-1640 without doxorubicin the day before violacein treatment.

#### 2.2.4 Human Serum Differentiated Huh7

Huh7 cells were differentiated in 2% human serum (PAN-Biotech #P40-2701) for three weeks, as described previously (25, 26).

#### 2.2.5 Isolation, Cultivation, and Polarization of Human Monocyte-Derived Macrophages (HMDMs)

Buffy coats from healthy donors were collected from the Blood Donation Center, Saarbruecken, Germany, authorized by the local ethics committee (State Medical Board of Registration, Saarland, Germany; permission no. 173/18). Lymphocyte Separation Medium 1077 (PromoCell #C-44010) was used to isolate peripheral blood mononuclear cells (PBMC) in Leucosep tubes (Greiner #227290) using density gradient centrifugation. Positive selection was performed using CD14 magnetic beads (Miltenyi #130-050-201) to sort PBMCs for CD14 positive cells. Upon sorting, monocytes at a density of 0.5×10^6^ cells/ml were seeded and differentiated in RPMI-1640 supplemented with 20 ng/ml human recombinant macrophage colony-stimulating factor (M-CSF, Miltenyi #130-096-492) for 6 days.

Polarization of HMDMs was performed *in vitro*: To obtain M1 polarization, media was supplemented with 20 ng/ml recombinant IFNγ (Miltenyi #130-096-484) and 100 ng/ml LPS (Ultrapure LPS from *Escherichia coli* K12 #tlrl-peklps), while either 20 ng/ml IL4 (Miltenyi #130-093-921) or IL10 (Miltenyi #130-093-948) was used to obtain M2 polarization. Differentiation media without any further supplementation was used to attain M0 macrophages. In all experiments comparing macrophage subsets, cells from the same donor were used.

#### 2.2.6 Isolation and Cultivation of Bone Marrow-Derived Macrophages (BMMs)

BMMs were obtained from wildtype or *Nlrp3* knockout C57B/6 mice and cultivated as described previously (27). Cells were seeded per well into 96 well plates (75,000 per well) for MTT assays and into 12 well plates (500,000 per well) for RT-qPCR. The use of murine BMMs was approved by the local animal welfare committee (Landesamt für Verbraucherschutz, Saarbrücken, Germany; AZ.: GB 3-2.4.2.2.-/2016 and AZ: 2.4.2.2.-06/2020) and was in accordance with the European Legislation on Protection of Animals (Guideline 2010/63/EU) and the NIH Guidelines for the Care and Use of Laboratory Animals (AZ: 39/3.5.2.1).

Polarization of BMMs was performed *in vitro*: To obtain M1 polarization, media was supplemented with 20 ng/ml recombinant IFNγ (Biomol #87389.100) and 100 ng/ml LPS (Ultrapure LPS from *Escherichia coli* K12 #tlrl-peklps), while either 20 ng/ml IL4 (Miltenyi #130-094-061) or IL10 (Miltenyi #130-094-067) was used to obtain M2 polarization. Polarization was performed in the absence or presence of violacein. Differentiation media without any further supplementation was used to attain M0 macrophages. In all experiments comparing macrophage subsets, cells from the same donor were used.

### 2.3 qPCR

RNA was isolated using the High Pure RNA Isolation Kit (Roche #11828665001), and reverse transcribed using the High Capacity cDNA Reverse Transcription Kit (Thermo Fisher Scientific #4368813) in the presence of an RNase inhibitor (Invitrogen #10777-019) according to the manufacturer’s instructions. cDNA was analyzed by qPCR using a 5xHotFirePol EvaGreen qPCR Mix (Solis BioDyne #08-24-00020) and the following primers: hu_*TNF*_for: 5´CTCCACCCATGTGCTCCTCA3´, hu_*TNF*_rev: 5´CTCTGCCAGGGGCTCTTGAT3´, hu_*IL1B*_for: 5´GGCTGCTCTGGGATTCTCTT3´, hu_*IL1B*_rev: 5´AGTCATCCTCATTGCCACTGTAA3´, hu_*IL6*_for: 5´ACATCCTCGACGGCATCTCA3´, hu_*IL6*_rev: 5´TCACCAGGCAAGTCTCCTCATT3´, hu_*IL10*_for: 5´CAACAGAAGCTTCCATTCCA 3´, hu_*IL10*_rev: 5´AGCAGTTAGGAAGCCCCAAG3´, mu_*Il1b*_for: 5´CCAAAAGATGAAGGGCTGCTT3´, mu_*Il1b*_rev:5´ GGAAGGTCCACGGGAAAGAC3´, mu_*Il6*_for: 5´AAGAAATGATGGATGCTACCAAACTG3´, mu_*Il6*_rev: 5´GTACTCCAGAAGACCAGAGGAAATT3´, mu_*Tgfb*_for: 5´ACCCTGCCCCTATATTTGGA3´, mu_*Tgfb*_rev: 5´CGGGTTGTGTTGGTTGTAGAG3´, mu_*Tnf*_for: 5´CCATTCCTGAGTTCTGCAAAGG3´, mu_*Tnf*_rev: 5´AGGTAGGAAGGCCTGAGATCTTATC3´. The PCR was performed in a CFX96 touch™ Real-Time PCR detection system (BioRad). Data was normalized to the housekeeping gene *RNA18S*.

### 2.4 Violacein

Violacein was produced using recombinant *E. coli* as described before (2). Then, cells were harvested and extracted twice with ethanol. Violacein was purified from the extract (>97%) (Bioviotica Naturstoffe, Göttingen, Germany). It was dissolved in DMSO because of its poor solubility in water. DMSO solvent controls were included in the experiments.

### 2.5 Endotoxin Detection

Prior to cell treatment, violacein was tested for the absence of endotoxins using the Endozyme^®^ II assay kit (Biomérieux #890030) according to the manufacturer’s instructions.

### 2.6 MTT Assay

Cells were seeded into 96 well plates in appropriate cell densities to attain confluency the next day. Cells were treated as indicated before media was removed, and 0.5 mg/ml MTT (3-[4,5-dimethylthiazol-2-yl]-2,5- diphenyltetrazolium bromide, Sigma–Aldrich) dissolved in the respective culture media was added. Cells were lysed in DMSO, and absorbance was measured at 560 nm using a microplate reader (GloMax™).

### 2.7 APH Assay

Spheroids were treated as indicated, and cell viability was assessed by acid phosphatase (APH) assay. For this, supernatants were replaced by 100 μl assay buffer (0.1 M sodium acetate (pH 5.2), 0.1% (v/v) Triton X-100, supplemented freshly with 4 mg/ml p-nitrophenyl phosphate (final pH 4.8, Thermo Fisher Scientific #34045). Spheroids were incubated at 37°C for 90 mins before 10 µl NaOH were added. Absorption was measured at 405 nm on a microplate reader (GloMax™).

### 2.8 Caspase 3/7 Activity

Cells were collected (adherent cells were trypsinized and detached cells in the supernatant were spun down), before they were washed with ice-cold PBS, and 70 µl lysis buffer were added (25 mM HEPES, 5 mM MgCl_2_, 1 mM EDTA, 0.1% [v/v] Triton X-100). Lysates were centrifuged (14,000*g*, 10 min, 4°C) and 10 µl of the supernatant were transferred to white 96 well plates. 90 µl substrate solution were added (55 µM of fluorogenic substrate Ac-DEVD-AFC (Enzo, #ALX-260-032-M005), 50 mM HEPES, 0.1% [w/v] CHAPS, 1% [w/v] sucrose, 10 mM DTT, pH 7.5). Free AFC (7-amino-4-trifluoro-methyl coumarin) was measured at 37°C in a microplate reader (GloMax™, excitation: 405 nm; emission: 495-505 nm). Data was normalized to protein concentrations as determined by a BCA assay.

### 2.9 ATP Release

50 µl supernatants were transferred to a white 96 well plate. Assay buffer was injected (50 µl; 20 mM Tricine, 1.1 mM MgCO_3_ Mg(OH)_2_, 2.7 mM MgSO_4_, 100 µM EDTA, 33.3 mM DTT, 0.213 mg/ml coenzyme A, 470 µM D-luciferin, 20 µg/ml luciferase), and luminescence was detected in a microplate reader (GloMax™).

### 2.10 ROS Production

The homovanillic acid oxidation assay was performed to detect produced hydrogen peroxide in a defined period. The HVA assay reaction solution (0.1 mM HVA, 4 U/ml HRP in PBS) was prepared freshly and was protected from light. The cells were washed with PBS before 150 µl assay solution were added. Plates were protected from light and incubated for 1 h at 37°C. Afterward, 100 µl supernatant were transferred into a white 96 well plate, and the reaction was stopped by adding 15 µl stop solution (0.1 M glycine, 0.1 M NaOH, 25 mM EDTA). Fluorescence was measured at 312 nm excitation and 420 nm emission.

### 2.11 Proliferation

PANC-1, Huh7, and HCT116 cells were seeded at an appropriate cell density to reach 10% confluency the next day. Cells were treated as indicated. Automated microscopy was performed using the IncuCyte^®^ S3 system. The quantification of the proliferation was calculated by the IncuCyte^®^ basic analyzer software based on cell confluency.

### 2.12 Migration

PANC-1, Huh7, and HCT116 cells were seeded into Image Lock 96-well plates at an appropriate cell density to reach 90% confluency the next day. The scratches were made using the WoundMaker™ tool (IncuCyte^®^ Migration Kit). Media without FCS was used to wash the cells and for further cultivation. Cells were treated as indicated. Automated microscopy was performed using the IncuCyte^®^ S3 system. The quantification of the migration was calculated by the IncuCyte^®^ software.

### 2.13 TLR Reporter Cell Assay

HEK-Blue™ hTLR4, HEK-Blue™ Null2, THP1-XBlue™, and RAW-Blue™ cells stably express a secreted embryonic alkaline phosphatase (SEAP) gene inducible by NF-κB and AP-1 transcription factors and are used to determine NF-κB/AP-1 activity. Tumor cells were seeded in 6 well plates in appropriate numbers to reach 90% confluency the next day. On the next day, tumor cells were treated with 50 μM violacein, oxaliplatin, or solvent control. After 4 h, the medium containing the respective treatment was removed, and wells were replaced with fresh media. 20 h later, supernatants were collected and centrifuged to remove dead cells. These dead tumor cell-conditioned media (dTCM) were then added to the reporter cells seeded a day prior (as per the manufacturer’s protocol). After 24 h, SEAP activity in the reporter cell supernatants was determined using the QUANTI-Blue™ solution according to the supplier’s instructions. Lipopolysaccharide (LPS-EK Ultrapure, In vivoGen) at a concentration of 100 ng/ml for RAW-Blue™ and 10 ng/ml in HEK-Blue™ hTLR4, HEK-Blue™ Null2, and THP1-XBlue™ were used as a positive control in all reporter cell assays. SEAP levels were determined at 600 nm with a microplate reader (GloMax™) and normalized to cell confluency as determined by IncuCyte^®^ analysis.

### 2.14 Macrophage Morphology Analysis

*In vitro* differentiated and polarized macrophages were treated with the dTCM also used for reporter cell analysis. The IncuCyte^®^ S3 system was used to image the cells at the beginning and the end of treatment. Cells were analyzed for their morphology using the IncuCyte^®^ cell-by-cell analysis software and grouped on the basis of their eccentricity into either round or elongated shapes.

### 2.15 NO Production

RAW264.7 cells were cultured in 96-well plates and treated with violacein for 1 h before adding 1 µg/ml LPS. After 20 h, nitrite as a metabolite of NO was measured by Griess reaction as described previously (28).

### 2.16 Statistical Analysis

Data analysis was done by Microsoft Excel, and statistical analysis was performed using OriginPro^®^ 2019. Based on group number, statistical differences were calculated using Student’s t-test for two groups and one-way ANOVA with Tukey’s or Bonferroni’s *post hoc* analysis for more than two groups. IC_50_ values were calculated using nonlinear regression analysis. Experiments were performed in at least three independent experiments, and data are represented as means ± SEM if not indicated otherwise. * p < 0.05; ** p < 0.01; *** p < 0.001.

## 3 Results

### 3.1 Activity of Violacein Against Different Hallmarks of Cancer

**Figure 1:**
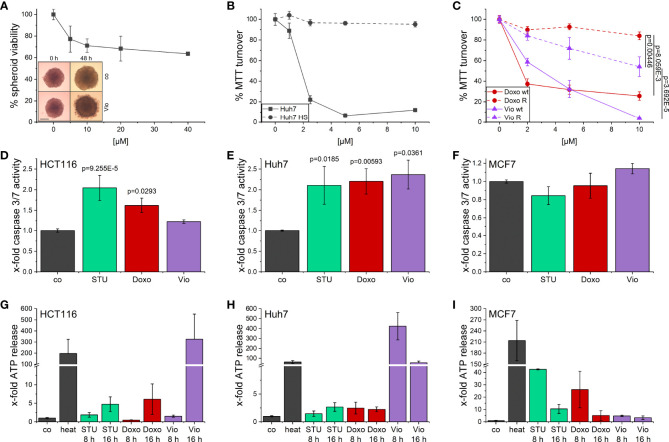
Cytotoxic effects of violacein against cancer cells. **(A)** HCT116 spheroids were treated with violacein or DMSO solvent control for 48 h before spheroids were imaged and cellular viability was analyzed. Representative pictures for the DMSO control (co) and 20 µM violacein treatment are shown for the start and treatment endpoint; scale bar = 250 μm. **(B)** Standard Huh7 or human serum (HS)-differentiated Huh7 cells were treated with violacein or solvent control (set to 100%), and cells were analyzed in an MTT assay 48 h after treatment. **(C)** Doxorubicin resistant (R) and Huh7 wildtype (wt) cells were treated with violacein (Vio) or doxorubicin (Doxo), and cells were analyzed by MTT assay 48 h after treatment. **(D-F)** Caspase 3/7 activity of **(D)** HCT116, **(E)** Huh7, and **(F)** MCF7 cells treated for 8 h with staurosporine (STU, 1 µM), doxorubicin (5 µM), or violacein (10 µM), respectively. **(G-I)** Luciferase-based measurement of ATP release to the supernatant of **(G)** HCT116, **(H)** Huh7, and **(I)** MCF7 cells treated for 8 h or 16 h with staurosporine (STU, 1 µM), doxorubicin (Doxo, 5 µM), or violacein (Vio, 10 µM), respectively. D-I: Means values for solvent-control treatment were set as 1.

The activity of violacein against cancer cells was first assessed using an MTT assay after 48 h treatment. In a set of cell lines derived from various tumor entities, violacein exerted toxic effects in the low micromolar range, with IC_50_ values ranging from 0.393 µM in SK-MEL-5 cells to 9.864 µM in HepG2 cells (**Supplementary Table 1**). These cytotoxic effects could also be observed when HCT116 cells were grown in a 3D tumor spheroid model. In this model, violacein treatment not only inhibited the growth of the spheroid but also resulted in a detachment of the outer cells from the core (**Figure 1A**). To test whether violacein affects normal cells to the same extent, we used a model of long-term cultivation of Huh7 in human serum. The transition of the cell line towards a more normal, hepatocyte-like phenotype has been demonstrated previously (25, 26). In this model, the activity of violacein was abolished (**Figure 1B**). Moreover, violacein was able to kill doxorubicin-resistant Huh7 cells (**Figure 1C**).

For a clearer picture of provoked cell death, the activation of caspase 3/7 (**Figures 1D–F**) as central players in programmed cell death and the release of ATP (**Figures 1G–I**) were examined. In Huh7 cells, 8 h treatment with violacein resulted in an activation of caspase 3/7 comparable to the controls staurosporine (STU) and doxorubicin (Doxo) (**Figure 1E**), which was not the case in HCT116 (**Figure 1D**). In MCF7 cells, which do not express caspase 3, none of the treatments activated caspase 7 (**Figure 1F**). In HCT116 cells, a release of ATP could only be observed at a later timepoint (**Figure 1G**), while Huh7 cells already released ATP after 8 h treatment (**Figure 1H**). In MCF7 cells, no ATP was detectable in the supernatant upon 16 h treatment (**Figure 1I**).

Since violacein has been demonstrated to increase the production of reactive oxygen species (ROS) in previous studies (5, 6), and excessive ROS levels can cause cell death, their production was measured in an homovanillic acid (HVA) assay. However, concentrations up to 2.5 µM did not provoke a significant ROS production (**Supplementary Figure 1**).

**Figure 2:**
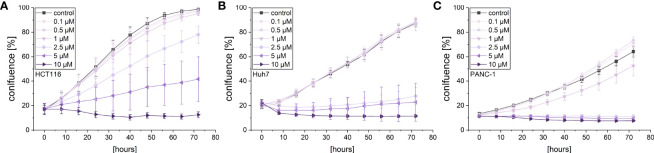
Live-cell microscopy-based analysis of the anti-proliferative effect of violacein. Cell confluency of **(A)** HCT116, **(B)** Huh7, and **(C)** PANC-1 cells was monitored in an IncuCyte^®^ S3 system during violacein or DMSO solvent control treatment over 72 h.

Given the cytotoxic activity of violacein against different cancer cell lines, its effects on cancer cell proliferation and migration were investigated in order to examine whether violacein also affects other hallmarks of cancer. The observation of cell proliferation in an automated microscopy approach revealed a dose-dependent inhibition of proliferation in HCT116, Huh7, and PANC-1 cells in concentrations below the IC_50_ for the respective cell line (**Figure 2**). As expected, at higher concentrations cell confluency went down confirming cytotoxic actions.

**Figure 3:**
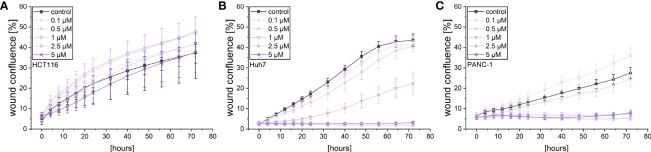
Live-cell microscopy-based analysis of the effect of violacein on cell migration. After wounding, wound confluence of violacein or DMSO solvent control-treated **(A)** HCT116, **(B)** Huh7, and **(C)** PANC-1 cells was monitored in an IncuCyte^®^ S3 system over 72 h.

A live cell microscopic approach was also used to monitor the effects of violacein on cellular migration in a scratch-wound assay. While violacein showed no significant action on HCT116 migration (**Figure 3A**), migration of Huh7 and PANC-1 cells was inhibited (**Figures 3B, C**).

### 3.2 Activity of Violacein on Macrophages

**Figure 4:**
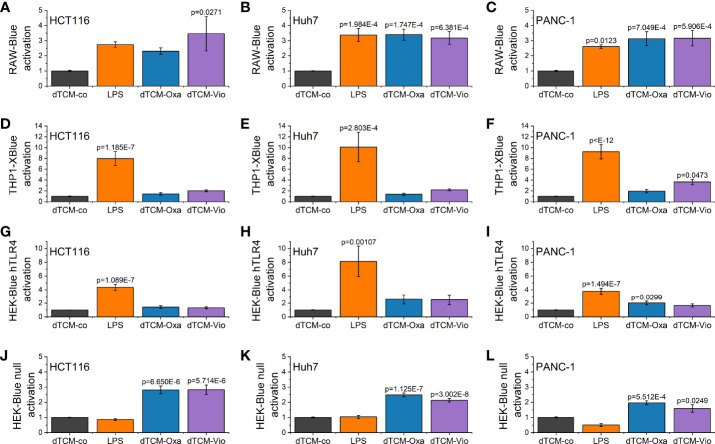
Macrophage activation by violacein-killed tumor cell supernatant. HCT116, Huh7, and PANC-1 cells were treated with 50 µM violacein or oxaliplatin for 4 h before the media was changed. Then, 24 h after treatment, supernatants from untreated (co) and compound-treated cells were collected, and dead cells were removed. **(A-C)** RAW-Blue™, **(D-F)** THP1-XBlue™, **(G, H)** HEK-Blue™ hTLR4, and **(J-L)** HEK-Blue™ Null2 reporter cells were incubated with the respective dead tumor cell-conditioned medium (dTCM, 50% v/v) or LPS (100 ng/ml for RAW-Blue™ and 10 ng/ml for THP1X-Blue™, HEK-Blue™ hTLR4, and HEK-Blue™ Null2) for 24 h. The activation of the reporter cells was determined by colorimetric QUANTI-Blue™.

To elucidate whether the supernatant of violacein-killed cells affects macrophage activation, RAW-Blue™ and THP1-XBlue™ reporter cells were incubated with the dead tumor cell-conditioned media (dTCM) of HCT116, Huh7, or PANC-1 cells killed by violacein (dTCM-Vio) or oxaliplatin (dTCM-Oxa) as a control. The toll-like receptor 4 (TLR4) agonist lipopolysaccharide (LPS) was used as a positive control of macrophage activation. In RAW-Blue™ cells, the addition of dTCM resulted in a potent inflammatory activation (**Figures 4A–C**). While the dTCM of HCT116 (**Figure 4D**) and Huh7 (**Figure 4E**) cells provoked only a tendency of an effect on THP1-XBlue™ cells, the PANC-1 dTCM-Vio also activated NF-κB in these cells (**Figure 4F**). Since the release of the TLR4 ligand HMGB1 is associated with an immunogenic nature of compound-induced cell death, the specific activation of TLR4 was investigated. While none of the dTCM-Vio activated HEK-Blue™ hTLR4 cells (**Figures 4G–I**), their parental cell line HEK-Blue™ Null, expressing TLR3, TLR5, and NOD1, but no TLR4, showed a response (**Figures 4J–L**).

**Figure 5:**
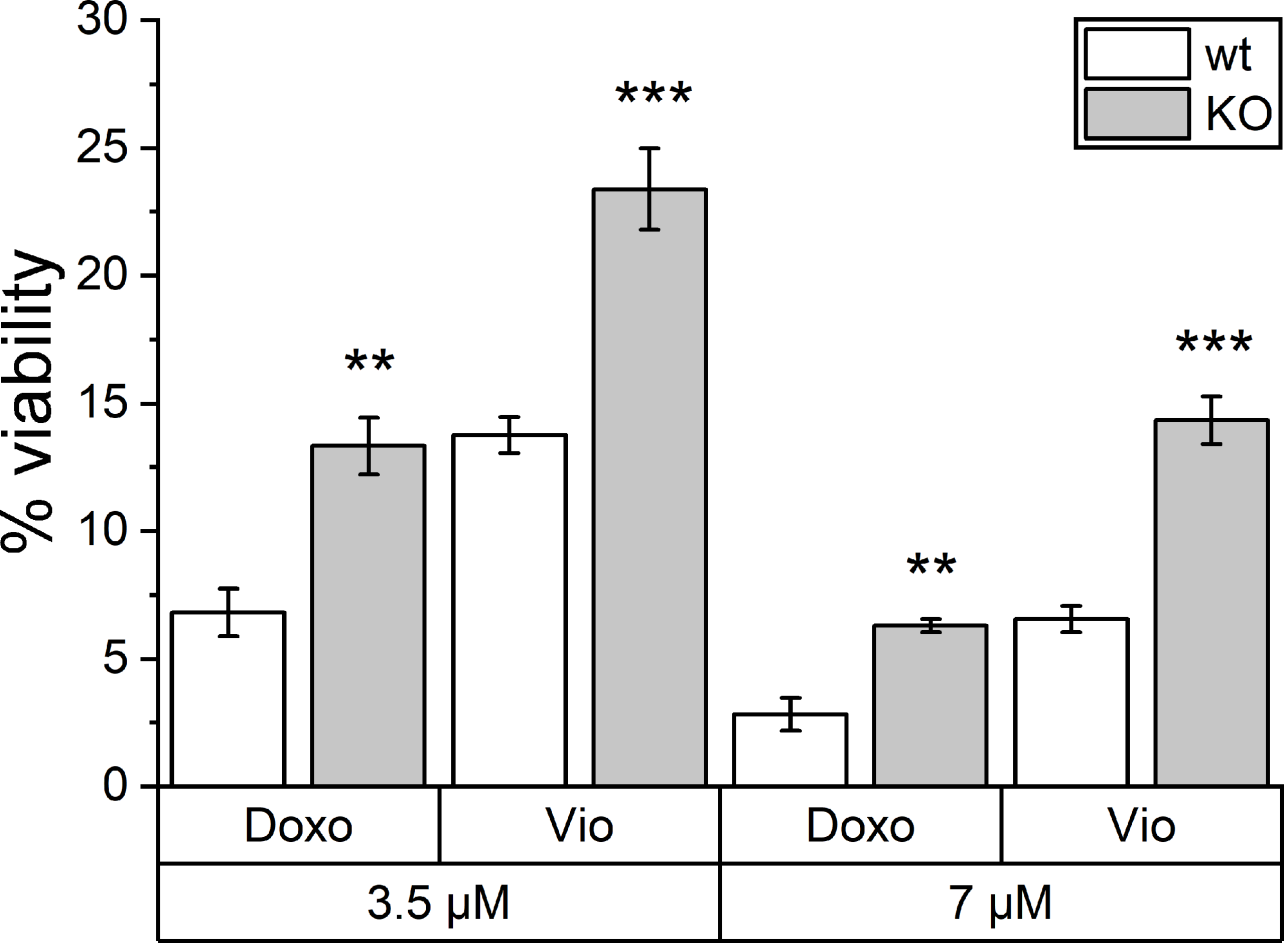
Toxic effects of violacein against Nlrp3 knockout macrophages. BMMs from wildtype (wt) and *Nlrp3* knockout (KO) mice were treated with violacein or doxorubicin, and cells were analyzed in an MTT assay 48 h after treatment. The viability of solvent control-treated wt and KO cells were set to 100%, respectively. ** p< 0.01, *** p<0.001.

Besides TLR4 signaling, another PRR family also contributes to ICD. NLRP3 inflammasome activation has been shown to be another key factor in ICD. Hence, violacein toxicity was investigated in *Nlrp3* knockout bone marrow-derived macrophages (BMMs). As assessed by MTT assays, violacein toxicity was significantly reduced in the knockout cells, as also observed for the ICD-inducer doxorubicin (**Figure 5**).

**Figure 6:**
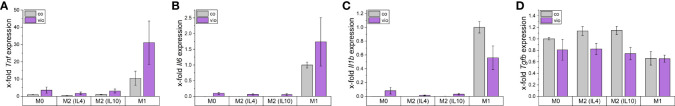
Macrophage gene expression after violacein treatment. BMMs were incubated with respective polarization stimuli alone or with 5 µM violacein for 24 h. Gene expression was determined *via* qPCR and values were normalized to *RnA18S* as a housekeeping gene and untreated **(A, D)** M0 or **(B, C)** M1 expression.

To assess the direct effects of violacein on macrophage activation, iNOS induction was analyzed by the determination of nitric oxide (NO) production using the Griess reagent. RAW264.7 cells were pre-treated with subtoxic concentrations for 1 h before LPS was added for stimulation. After 21 h no significant changes in the nitric oxide levels were observed (**Supplementary Figure 2A**). Moreover, violacein induced the expression of the inflammatory cytokines *Tnf*, *Il6*, and IL1b in M0 and M2 macrophages, while *Tgfb* was reduced (**Figure 6**). In inflammatory M1 macrophages, *Tnf* and *Il6* expression were further increased, while *Il1b* was decreased and *Tgfb* expression showed no alterations.

**Figure 7:**
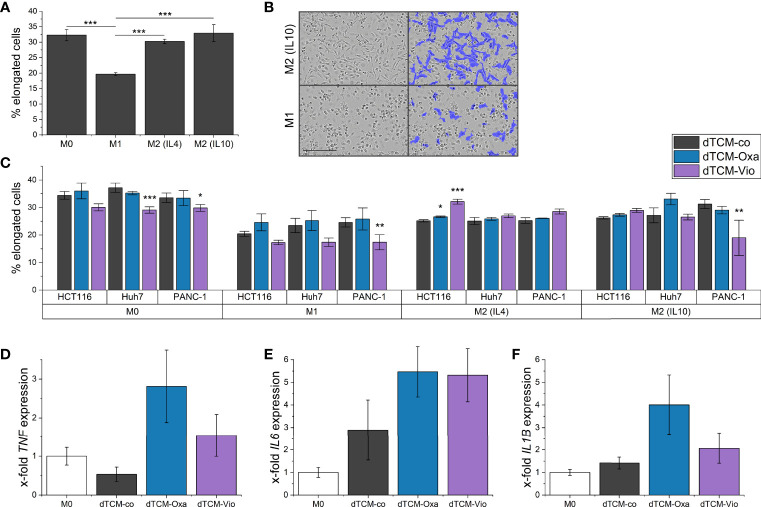
The supernatant of violacein-killed tumor cells affects macrophage phenotype. HMDMs were differentiated and polarized *in vitro* into M0, M1, M2 (IL4), and M2 (IL10) macrophages for 24 h. **(A)** Differently polarized macrophages were imaged using the IncuCyte^®^ S3 system, and cells were grouped based on their eccentricity in an elongated or round morphology using the IncuCyte^®^ cell-by-cell analysis software. **(B)** Left: Representative pictures of M1 and M2 (IL10) macrophages are shown. Right: The violet mask labels the cells detected by the software as elongated. Scale bar = 200 µm. **(C)** Polarized macrophages were treated with dTCMs (37.5% v/v) from either HCT116, Huh7, or PANC-1 treated with either oxaliplatin (Oxa), violacein (Vio), or solvent control (co). Macrophage morphology was analyzed 48 h after treatment. *n* = 2 (triplicates). **(D-F)** M0 macrophages were treated with dTCMs (37.5% v/v) from Huh7 cells for 24 h. Gene expression was determined *via* qPCR and values were normalized to *RNA18S* as a housekeeping gene; 2 (triplicates). * p< 0.05, ** p< 0.01, *** p<0.001.

To further elucidate the influence of dTCM-Vio on human macrophage polarization, their morphology was assessed by automated microscopy. As also seen in previous studies (29), human monocyte-derived macrophages (HMDMs) that were differentiated and polarized *in vitro* display distinct morphologies. While M1 macrophages have a higher proportion of round cells, M2 polarized cells are elongated (**Figures 7A, B**). Differently polarized HMDMs were treated with dTCMs. In this setup, primarily dTCM-Vio from PANC-1 cells decreased the proportion of elongated cells for M0, M1, and M2 (IL10) macrophages (**Figure 7C**). This was also observed for M0 macrophages incubated with dTCM-Vio from Huh7 cells, while the HCT116 supernatant increased the number of elongated M2 (IL4) cells. Moreover, dTCM-Oxa and dTCM-Vio from Huh7 cells increased the expression of *TNF* and *IL6* in M0 macrophages (**Figures 7D, E**), while only dTCM-Oxa induced *IL1B* (**Figure 7F**).

## 4 Discussion

The major aim of this study was to characterize the anti-cancer activities of violacein with a focus on the interplay of cancer cells with macrophages of their tumor microenvironment. In line with previous findings, our data show that violacein demonstrates substantial anti-cancer activities targeting different hallmarks of cancer.

The low micromolar IC_50_ values demonstrate potent activity against a variety of tumor entities. However, differentiated Huh7 cells are significantly less affected. This finding further supports the low toxicity found *in silico* and *in vivo* in previous studies (9, 30). One possible explanation is that differentiated and growth-arrested cells are less susceptible to the mode of action of violacein. The observed anti-proliferative effect even at subtoxic concentrations supports this suggestion. A reduced proliferation upon violacein treatment was demonstrated before in melanoma cells *via* AKT inhibition (31), and ERK inhibition in head and neck cancer shown *in vitro* and *in vivo* (32). Our data show that subtoxic concentrations of violacein also inhibit tumor cell migration. The inhibition of the matrix metalloproteinases MMP2 and MMP2 activation, which play an essential role in tumor metastasis, has been demonstrated before (33).

The relationship of ROS with violacein-mediated actions is contradictory. While violacein was reported to induce ROS production in several studies (5, 32), we and others could not find a significant increase (8, 34). However, the use of different cell lines, treatment times, and detection methods could explain this observation.

Interestingly, violacein was able to efficiently eliminate hepatoma cells resistant against doxorubicin. The observed cytotoxicity against different cancer cells was further associated with a caspase 3/7 activation and a release of ATP in HCT116 and Huh7 cells. On the other hand, MCF7 cells, deficient in caspase 3, released no ATP under assay conditions. Previous studies found that violacein induces apoptosis and interferes with autophagy, as violacein-induced cell death was associated with apoptotic markers such as caspase 3 and PARP activation, BCL2 and BAX modulation, increase in p53/*TP53*, but also with augmented LC-II and SQSTM1/p62 levels (5, 6, 31, 32).

As anti-inflammatory actions of violacein were described *in vivo* (9), Venegas et al. extensively investigated its effects on cytokine production *in vitro* (10). In contrast to the *in vivo* data, they found an induction of the pro-inflammatory cytokine TNF in murine macrophages and IL6 and IL1β secretion in human peripheral blood mononuclear cells (PBMCs). These findings are in line with the elevated gene expression of *Tnf*, *Il6* and *Il1b* we observed in BMMs. Interestingly, Venegas et al. found different responses towards violacein between RAW264.7 macrophages and primary BMMs regarding cytokine expression, i.e., elevated cytokine expression in RAW264.7 but no effect on BMMs. Moreover, they found that the inflammatory macrophage activation was depended on TLR8 but not TLR7 receptor signaling.

To elucidate if violacein has other effects beyond actions on tumor-centric hallmarks of cancer, we employed NF-κB reporter cells to investigate the immunogenicity of violacein-induced cell death. To exclude direct effects of the compound as far as possible, the supernatant of the treated cells was replaced after 4 hours. This method of collecting tumor antigens and damage-associated molecular patterns (DAMPs) released from dying cells was used previously for an analysis of the immunogenicity of specific ssRNA molecules (35). Using this setup, our data provide first insights into the immunogenicity of violacein-induced cell death. The DAMPs released by the tumor cells upon violacein treatment showed strong activation of RAW-Blue™ cells, and a tendency was also observed in THP1-XBlue™ cells. Oxaliplatin as a well-established ICD inducer was used as a control (36). Since both cell lines express TLR4 and the TLR4-agonist HMGB1 is described as a prototypic ICD marker, the specific TRR4-dependent signaling was investigated. The explicit TLR4 activation could not be observed under the applied assay conditions. However, because the activated HEK-Blue™ Null2 reporter cells lack TLR8, it can be assumed that the observed effects in RAW and THP1 reporter cells are not due solely to the already known TLR8 activation.

Besides TLR4 signaling, another PRR family also contributes to ICD. The activation of cytosolic NLRP3, e.g., by ATP, initiates the assembly of the inflammasome (37, 38), and mice deficient for the inflammasome component genes *Nlrp3* and *Casp1* failed to respond in an immunogenic fashion post-treatment with ICD inducers (39). Very interestingly, in this study, BMMs isolated from *Nlrp3* knockout mice were also less sensitive towards the ICD-inducer doxorubicin as well as towards violacein.

Moreover, *in vitro* differentiated and differently polarized macrophages treated with dTCM-Vio from different cell lines changed their morphology towards an M1-like, and therefore rather tumor-suppressing, phenotype. This shift in polarization was further supported by an increased inflammatory gene expression.

Altogether, our data reveal the natural compound violacein as a potent anti-cancer compound targeting diverse tumor-centric hallmarks of cancer. Moreover, the activities induced by violacein-mediated cell death might improve its efficiency *via* increased immunogenicity of the TME.

## Data Availability Statement

The original contributions presented in the study are included in the article/supplementary material, further inquiries can be directed to the corresponding author.

## Author Contributions

Initiation and direction of the study: AK. Conception and design of the study: CD and AK. Investigation and formal analysis: CD, SC, JH, and HS. Supervision: CD and AK. Providing violacein: MK and CW. Writing – original draft: CD, SC, and AK. Writing – review and editing: JH, HS, and CW. All authors contributed to manuscript revision, read, and approved the submitted version.

## Funding

This project was funded, in part, by the Deutsche Forschungsgemeinschaft (DFG, #KI702) and the Young Investigator Grant of Saarland University. We acknowledge support by the DFG and Saarland University within the funding programme Open Access Publishing.

## Conflict of Interest

The authors declare that the research was conducted in the absence of any commercial or financial relationships that could be construed as a potential conflict of interest.

## Publisher’s Note

All claims expressed in this article are solely those of the authors and do not necessarily represent those of their affiliated organizations, or those of the publisher, the editors and the reviewers. Any product that may be evaluated in this article, or claim that may be made by its manufacturer, is not guaranteed or endorsed by the publisher.
