# Supplementary material for: Characterization of Anti-Cancer Activities of Violacein: Actions on Tumor Cells and the Tumor Microenvironment
Source: Front Oncol. 2022 May 11;12:872223. doi: 10.3389/fonc.2022.872223 (PMC9130777; doi:10.3389/fonc.2022.872223)
Supplement: Supplementary file 1 [file DataSheet_1.pdf]

Supplementary Material

Characterization of anti-cancer activities of violacein: Actions on tumor cells and the tumor microenvironment

Charlotte Dahlem[1], Shilpee Chanda[1], Jan Hemmer[1], Hanna Schymik[1], Michael Kohlstedt[2], Christoph Wittmann[2], Alexandra K. Kiemer[1]*

[1] Pharmaceutical Biology, Department of Pharmacy, Saarland University, 66123 Saarbruecken, Germany

[2] Institute of Systems Biotechnology, Saarland University, 66123 Saarbruecken, Germany

*** Correspondence:**
Alexandra K. Kiemer, Ph.D.
pharm.bio.kiemer@mx.uni-saarland.de

Cell line	Violacein (µM)
SK-MEL5	0.39
SW620	0.62
A549	0.66
HeLa	1.00
PANC-1	1.44
Huh7	1.88
MCF-7	1.89
CC-SW-1	2.89
HCT116	8.22
HepG2	9.86

Supplementary Table 1. IC_{50} values of violacein against cancer cell lines after 48 h treatment, as determined by MTT assays.

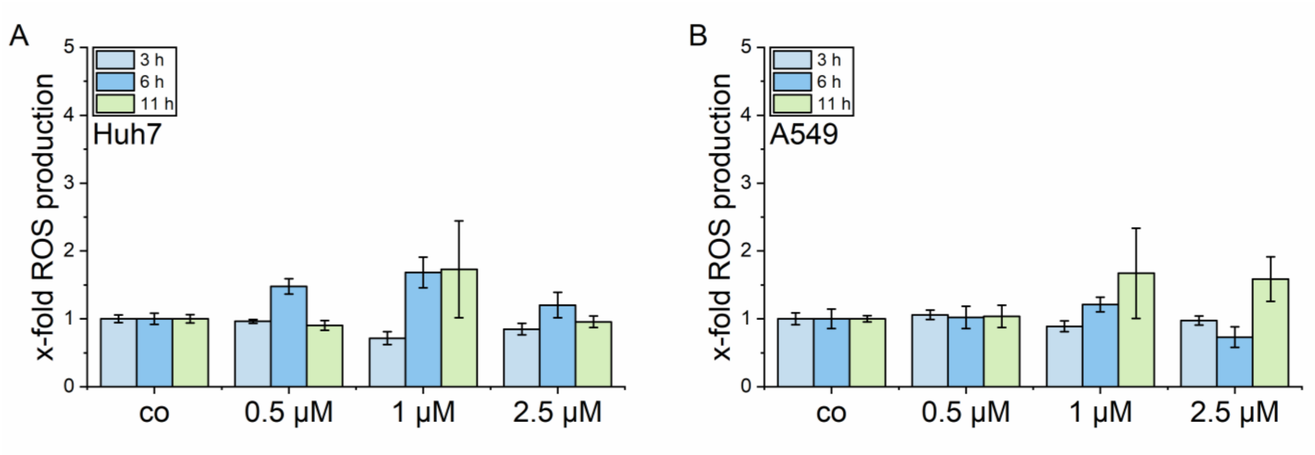

Supplementary Figure 2. ROS production after violacein treatment. ROS production was measured in an HVA assay 3, 6, or 11 h after treatment with violacein or DMSO solvent control. Data are normalized to protein amounts in each well and the DMSO control treatment at the respective time point.

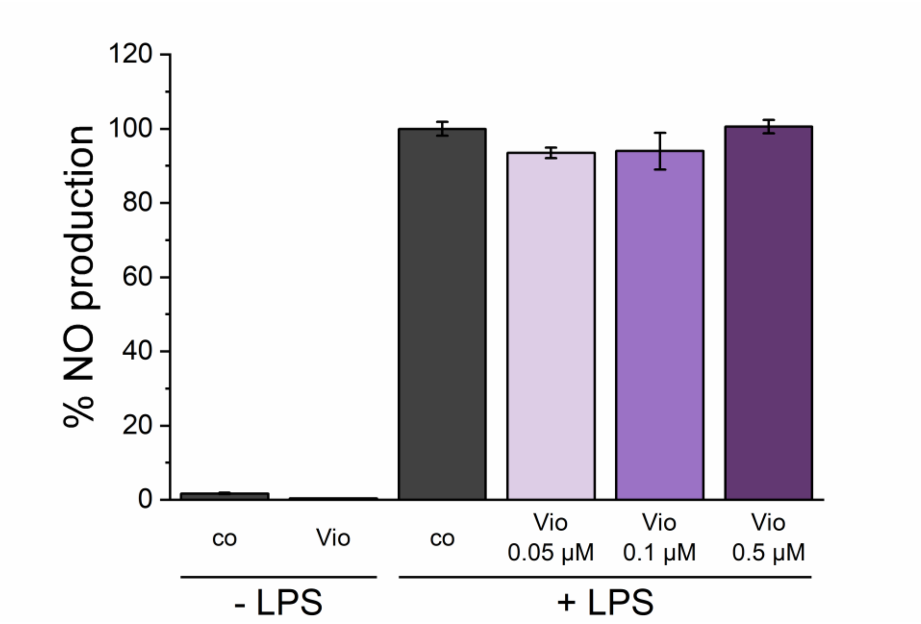

Supplementary Figure 2. NO production after violacein treatment. RAW264.7 cells were treated with violacein for 1 h before 1 µg/ml LPS was added. 20 h later, NO production was measured by a Griess assay and normalized to control-treated and LPS-stimulated cells. *n* = 1 (quadruplicates).
